# Junctional microstructure of the non-sticky spiral scaffold in the golden orb web spider, *Trichonephila clavata*

**DOI:** 10.1186/s42649-025-00114-6

**Published:** 2025-07-17

**Authors:** Seung-Min Lee, Myung-Jin Moon

**Affiliations:** Department of Biological Sciences, Dankook University, Cheonan, 31116 Korea

**Keywords:** Microstructure, Non-sticky spiral, Scaffold, Spider, Web

## Abstract

The non-sticky spiral silk, which typically serves as a temporary structural component in most orb-weaving spiders, functions as a permanent scaffold in the golden orb-web spider (*Trichonephila clavata*). Composed of double strands approximately 4 μm in diameter, the non-sticky spiral forms robust extended junctions exceeding 200 μm in radius. The muscular cell layer observed within the pyriform gland facilitates the active extrusion of pyriform fibers and cement, enabling efficient wrapping at the junctions. These robust junctions stand in stark contrast to the loose, droplet-mediated adhesion seen in sticky spirals, allowing the non-sticky spiral to enhance web stability and effectively prevent damage expansion. Furthermore, the non-sticky spiral plays an important role in localized web repair by replicating the original web's loop patterns to restore damaged areas. These findings suggest that the non-sticky spiral stabilizes the wide intervals between radii in the lower hub region, providing enhanced resistance to external forces and repairing structural damages. The results also demonstrate the evolutionary significance of utilizing non-sticky spiral as a permanent component, facilitating the construction and maintenance of large, densely structured orb webs.

## Introduction

Non-sticky spiral silk, a type of silk produced by the minor ampullate gland located in the spider's abdomen, is known to play a crucial role as an auxiliary scaffold during web construction (Römer and Scheibel 2008). Previous studies have reported that minor ampullate silk functions alongside major ampullate silk in forming radius and safety lines in orb webs (Hu et al. 2006).

In most orb-weaving spiders, non-sticky spiral serves as temporary scaffold for construct sticky spiral and is subsequently removed during the web construction process (Foelix 2011). However, in Trichonephila (Nephilidae), non-sticky spiral remains as a permanent structural component of the web (Hesselberg and Vollrath 2012; Zschokke and Vollrath 2013).

Non-sticky spiral consists of protein-based fibers, known as minor ampullate spidroins (MiSps), which provide both high tensile strength and flexibility (Guinea et al. 2012). These spidroins are characterized by repetitive sequences of glycine and alanine that form β-sheet structures, contributing to the silk’s mechanical properties (Vollrath and Knight 2001; Dicko et al. 2004).

The minor ampullate gland shares compositional similarities with the major ampullate gland, differing primarily in size and silk thickness (Foelix 2011; Andersson et al. 2013). While major ampullate silk has garnered significant attention for its unique mechanical properties, particularly in biomedical and materials science, research on non-sticky spiral remains relatively underdeveloped (Vollrath and Knight 2001).

Although limited to the mechanical properties of silk, Guinea et al. (2012) revealed that non-sticky spiral exhibits high flexibility and tensile strength, evolutionarily conserved in species such as Nephila and Argiope. Notably, non-sticky spiral demonstrates unique mechanical resilience by retaining its elasticity even in aqueous environments, highlighting its distinction from major ampullate silk. Nakamura et al. (2023) suggested that non-sticky spiral in the orb web spider, *Araneus ventricosus and Trichonephila clavata* exhibits higher water resistance and flexibility compared to major ampullate silk, potentially contributing to the structural integrity of the web.

Nonetheless, studies directly analyzing the microstructural morphology of natural web silk structures have yet to be reported. To bridge this gap, this study focuses on investigating the microstructural characteristics and adhesive interactions of non-sticky spiral with major structural silks in the golden orb web spider, *T clavata,* using electron microscopy techniques. This research provides new insights into the structural integration and functional role of non-sticky spiral within natural orb web framework.

## Materials and methods

Adult females of the golden orb web spider, *Trichonephila clavata* were collected in a local area near the Dankook University, Chungnam, Korea. Spiders were maintained in an indoor condition with natural lighting. To support orb web construction, a designated area (height × length × width, 2 m × 2 m × 50 cm) was set up on the floor and walls. This environment promoted the formation of complex orb webs. All spiders were fed insect larvae and water daily. After analysis, they were released back into the wild. The area of web sections damaged by intentional manipulation and prey capture was calculated. The repair of damaged sections typically occurred once every two days. Measurements were conducted in five individual spiders. Each spider was observed during three repair events.

Scanning electron microscopy (SEM) was used to observe the natural adhesion patterns of web silks. Stubs with double-coated conductive adhesive tape (Labsmro, Daejeon, Korea) were carefully applied to the plane of the orb web. To minimize deformation by unintended tension, the edges of the web sample were trimmed with beauty scissors immediately after contact with the tape.

The sample was coated with platinum-palladium with a thickness of 20 nm, using a Hitachi E-1030 ion sputter coater (Hitachi Co., Tokyo, Japan). Coated samples were observed with a Hitachi S-4300 (Hitachi Co., Tokyo, Japan) field emission scanning electron microscopy (FESEM) with an accelerating voltage of 5–20 kV (Lee and Moon 2025).

To investigate the adhesion mechanisms of non-sticky spiral within the web structure, specimens were carefully anesthetized for 3 min using pure carbon dioxide. Subsequently, the specimens were gradually cooled to 4 °C and dissected under a light microscope in a drop of spider Ringer’s solution. This solution comprised 160 mM NaCl, 7.5 mM KCl, 4 mM CaCl2, 1 mM MgCl2, 4 mM NaHCO3, 20 mM glucose, with a pH of 7.4. The anterior spinneret and pyriform silk gland were carefully extracted.

For transmission electron microscopy (TEM) analysis, the tissues were fixed in a solution containing 2% paraformaldehyde and 2.5% glutaraldehyde in phosphate buffer. Post-fixation was carried out using 1% osmium tetroxide in the same buffer solution. The sample underwent dehydration in a graded ethanol series followed by propylene oxide treatment before being embedded in EM-Bed 812 medium (Lee and Moon 2023). Semithin sections were stained with toluidine blue for histological observation. Ultrathin sections were prepared using an LKB ultramicrotome, then double-stained with uranyl acetate and lead citrate. The sections were examined under a transmission electron microscope (JEM 100 CX-II, JEOL, Tokyo, Japan) at an operating voltage of 80 kV.

## Results

**Fig. 1:**
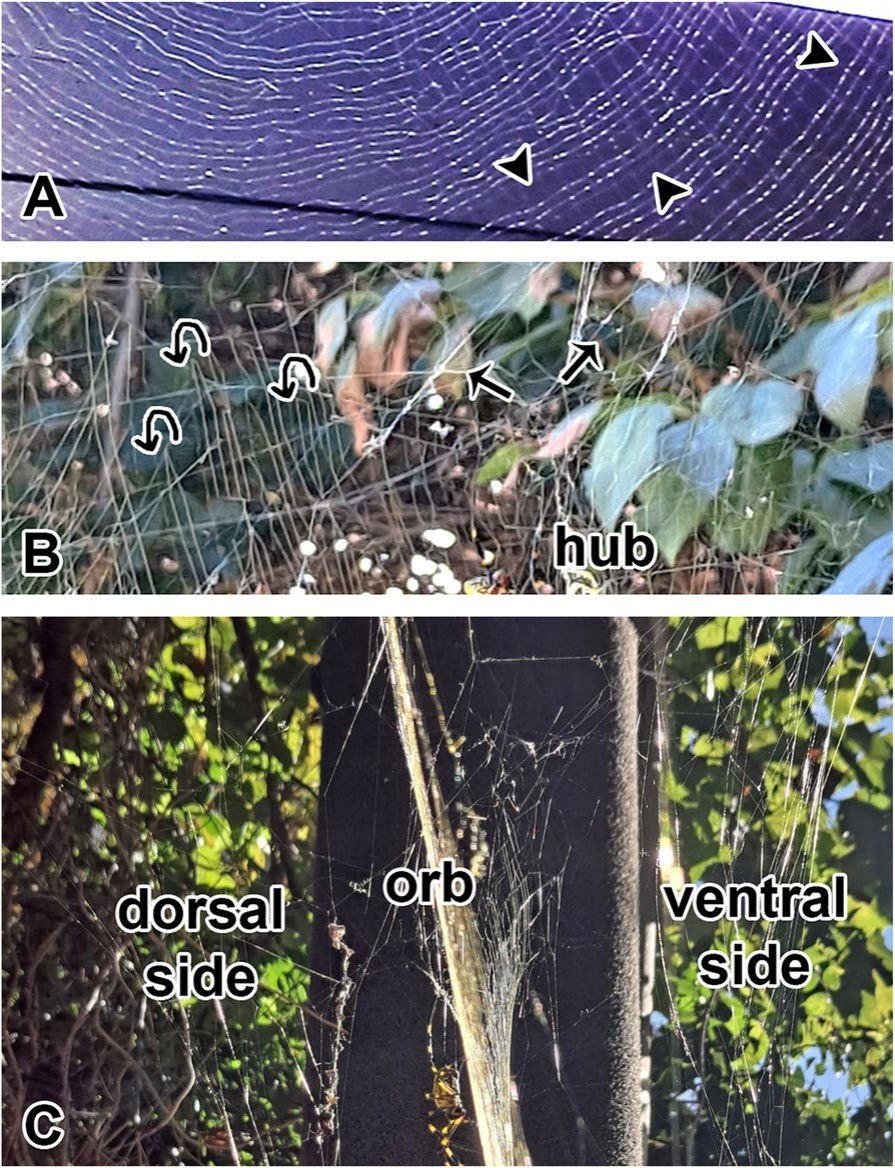
Structural characteristics of the hoof-shaped orb web in the golden orb web spider, *Trichonehpila clavata.* **A** The hub at the center of the orb web is displaced upward, resulting in an overall hoof-shaped configuration. Sticky spirals are almost parallelly arranged between the transparent non-sticky spiral (arrowhead). **B** The hoof-shaped web lacks spiral silk along the upper section. An irregular structural silk is arranged within a specific circular sector range (straight arrows) at the top to enhance the web’s stability. Furthermore, the spiral silk is organized in a looping manner (looping arrows). **C** Although the web is a two-dimensional structure, it is reinforced by auxiliary, irregular threads on both sides

The hub at the center of the orb web of the golden orb web spider, *Trichonephila clavata*, is distinct from other orb-weaving spiders, being displaced upward and forming a hoof-shaped structure (Fig. 1A). Additionally, the non-sticky spiral is not removed during the final stage of web construction. As a result, several strands of spiral silk are arranged between the non-sticky spiral, creating a pattern resembling a musical score. The upper section of the hub lacks spiral silk (Fig. 1B).

Instead, irregular structural silk is arranged within a specific circular sector at the top. Consequently, the spiral silk is not organized in a continuous spiral but forms loops. Although the web is a two-dimensional structure, it is reinforced by auxiliary, irregular silk threads on both sides (Fig. 1C). These side structures provide three-dimensional support, enhancing the web’s stability. The dorsal side of the spider is typically covered with organic debris, such as remnants of prey, leaves, and molted exoskeletons, whereas the ventral side has relatively less debris.

**Fig. 2:**
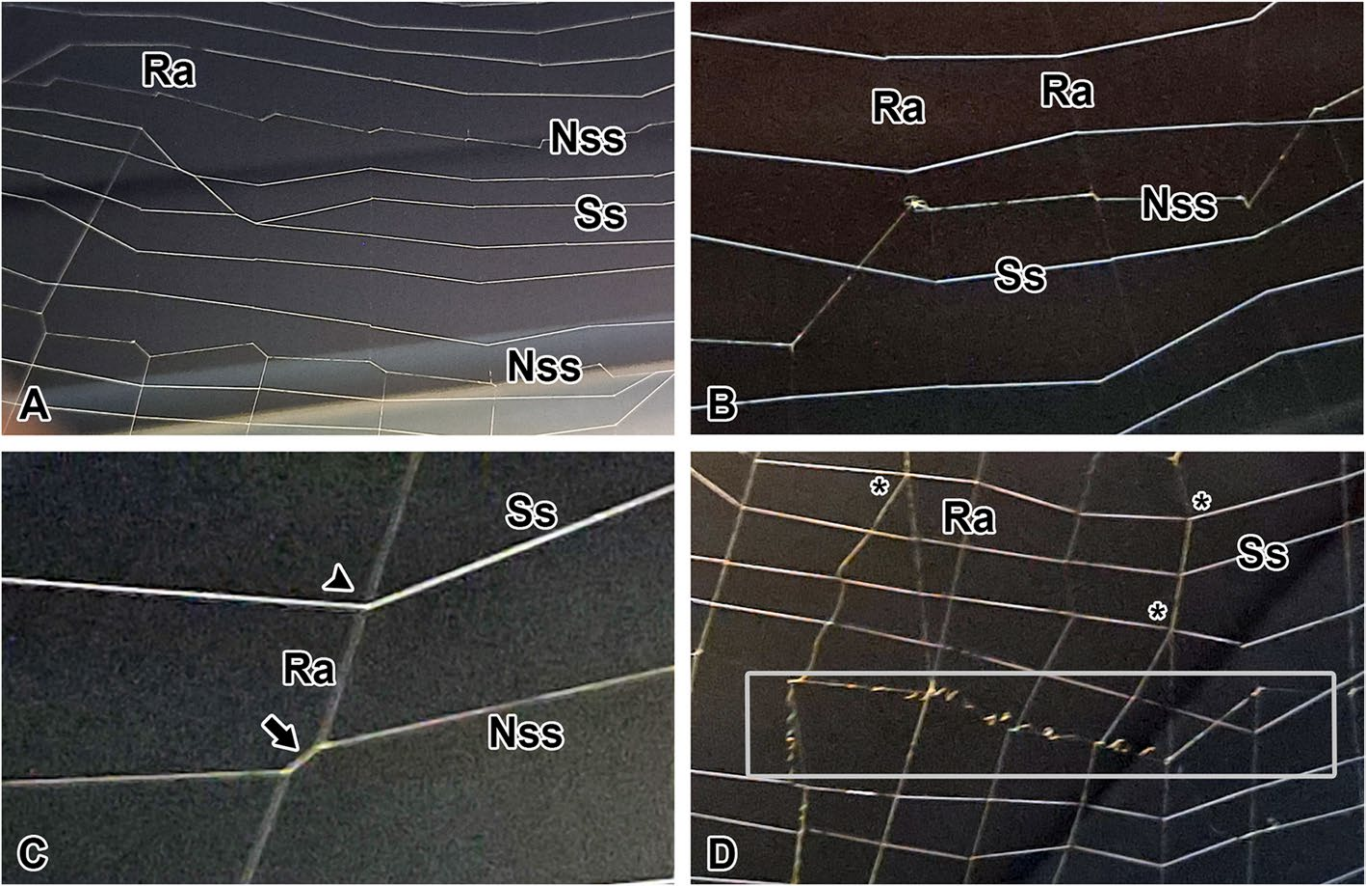
Web structure and junction of the non-sticky spiral*.* **A** The non-sticky spiral (Nss) intersects with the radii (Ra), forming grid-like sections within the orb web, where multiple strands of sticky spiral are arranged. Ss: sticky spiral. **B** Both types of spirals can be arranged in a spiral pattern centered around the web but may intersect at finer scales. **C** The junctions between the radii and the sticky spiral are point-shaped (arrowhead), whereas the junctions between the radii and the non-sticky spiral form slips (arrow), resulting in a zigzag arrangement. **D** Gray box shows that web damage leads to tension being released and the formation of coiled structures, while in adjacent sections, it remains intact and robust The radii branch more frequently as they extend toward the edges of the web

The non-sticky spiral intersects with the radii, forming grid-like sections within the orb web, where multiple strands of sticky spiral are arranged (Fig. 2A). Both types of spirals can be arranged in a spiral pattern centered around the web but may intersect at finer scales (Fig. 2B). This suggests that the contribution of each spiral to filling the web may not be influenced by one another.

The junctions between the radii and the sticky spiral are point-shaped, whereas the junctions between the radii and the non-sticky spiral form slips, resulting in a zigzag arrangement (Fig. 2C). In some sections of the non-sticky spiral, damage leads to tension being released and the formation of coiled structures, while in adjacent sections, it remains intact and robust (Fig. 2D). The radii branch more frequently as they extend toward the edges of the web. This structure provides sufficient space for the radii to establish grid-like sections as they extend further away from the hub.

**Fig. 3:**
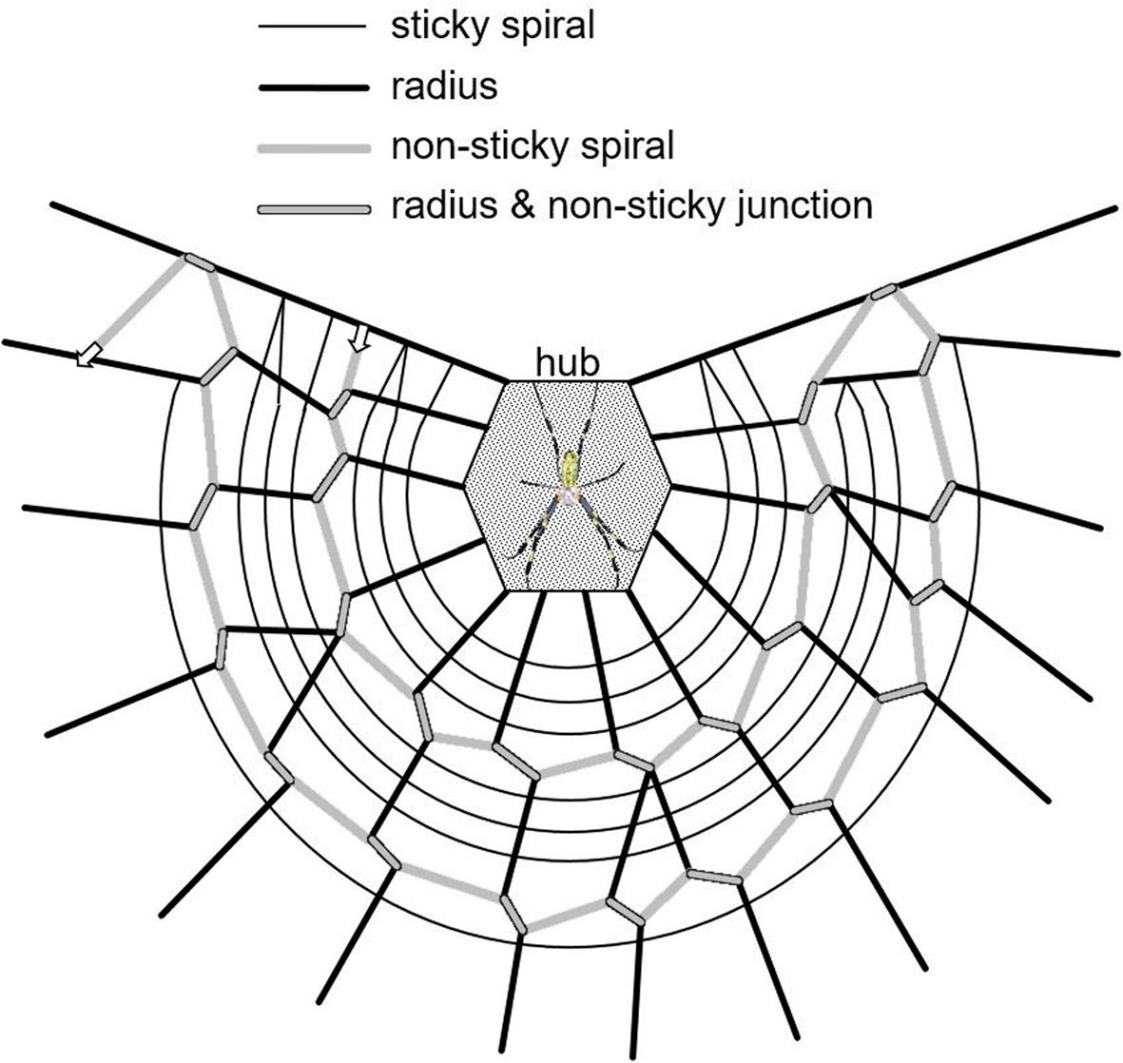
Illustration of hoof-shaped orb web*.* non-sticky spiral intersects with the radii, forming junctions that reinforce the framework. Arrows indicate the construction direction of the spiral as it loops across the structure

The hoof-shaped web is initially constructed by radii, which frequently branch out to form a strong lattice structure. Subsequently, a non-sticky spiral intersects with the radii, forming junctions that reinforce the framework (Fig. 3). It forms loops by spiraling outward from the center without surpassing the hub. Finally, a sticky spiral is meticulously constructed from the outer edge toward the center, filling the lattice space.

**Fig. 4:**
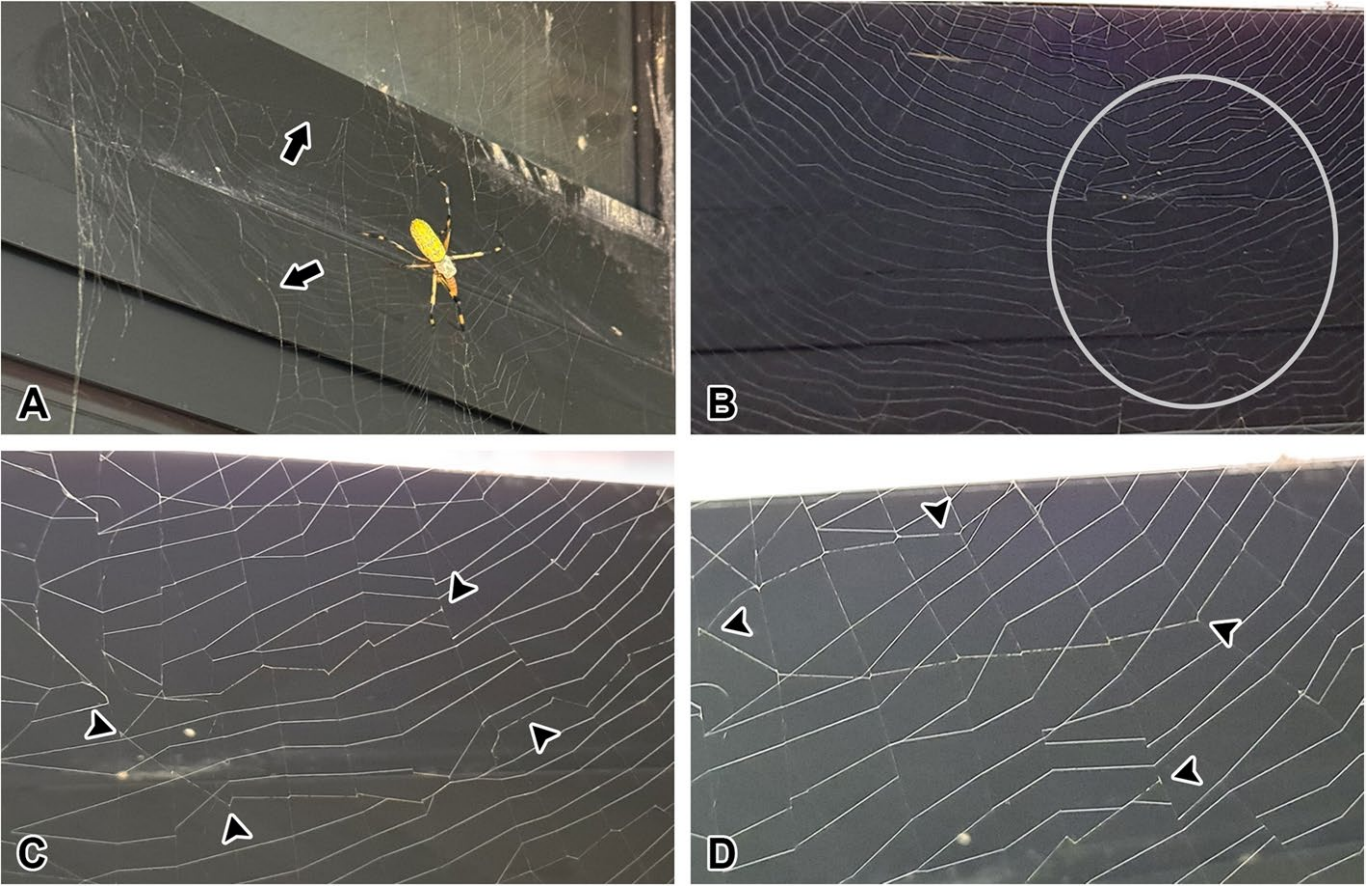
Localized web damage and repair mechanisms. **A** The web is damaged (arrow) due to collisions with prey or external factors, but only specific grid sections are affected, and the damage does not extend further, leaving the web largely intact. **B** The repaired web region (gray region) appears random in structure compared to the aligned grid arrangement of the undamaged region. **C** In the repaired web region, a localized non-sticky spiral is newly installed, spanning the area in a zigzag trajectory (arrowheads). **D** The repaired web region develops starting points and loops from the non-sticky spiral, which eventually become a permanent part of the web

The web is damaged due to collisions with prey or external factors, but only specific grid sections are affected, and the damage does not extend further, leaving the web largely intact (Fig. 4A). The repaired web region appears random in structure compared to the aligned grid arrangement of the undamaged region (Fig. 4B). In the repaired web region, a localized non-sticky spiral is newly constructed spanning the area in a zigzag trajectory (Fig. 4C). The repaired web region develops starting points and loops from the non-sticky spiral, which eventually become a permanent part of the web (Fig. 4D).

**Fig. 5:**
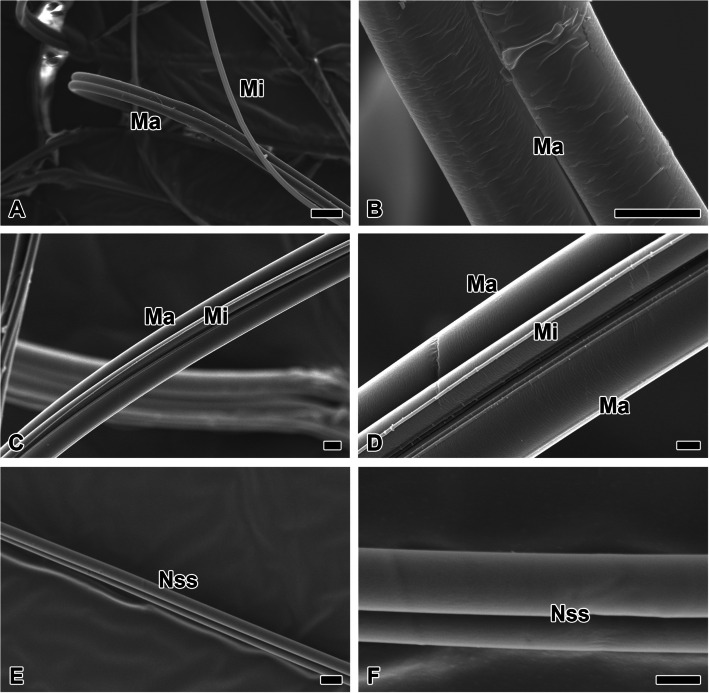
Fine structure of dragline silk co-spun from ampullate glands. **A** Dragline silk refers to the major ampullate silk (Ma) spun from silk precursors produced by the major ampullate gland. It is often co-spun with silk from the minor ampullate silk (Mi). **B** It is known that the silk from the major ampullate gland lacks any additional adhesive components. **C**, **D** When silk from both the major and minor ampullate glands is co-spun, the resulting fiber can consist of 3 to 4 strands, leading to enhanced tensile strength. **E** The non-sticky spiral is made of the same silk as the minor ampullate silk, with a diameter of approximately 3 μm and a smooth surface structure. **F** The surface shows no coating of adhesive substance. Each scale bar indicates 20 µm (A), 5 µm (B – C, E) and 2 µm (D, F), respectively

Dragline silk refers to the radius spinned from silk precursors in the major ampullate gland (Fig. 5A). In addition to the two strands of radius ejected from the anterior spinneret, minor ampullate silk can also be co-extruded. The silk produced by the major ampullate gland lacks any additional adhesive components on its surface (Fig. 5B).

When silk from the major and minor ampullate glands is co-spun, it forms a bundle consisting of 3–4 strands with enhanced tensile strength, used as a lifeline (Fig. 5C). The ampullate silk bundle is distinctly differentiated by thickness, with radius having a diameter of over 5 μm and minor ampullate silk measuring less than 5 μm (Fig. 5D). Meanwhile, the non-sticky spiral is also composed of minor ampullate silk (Fig. 5E). It has a diameter of approximately 3 μm and a smooth surface structure, but it is spun alone and consists of only two strands (Fig. 5F). Its surface lacks any adhesive coating.

**Fig. 6:**
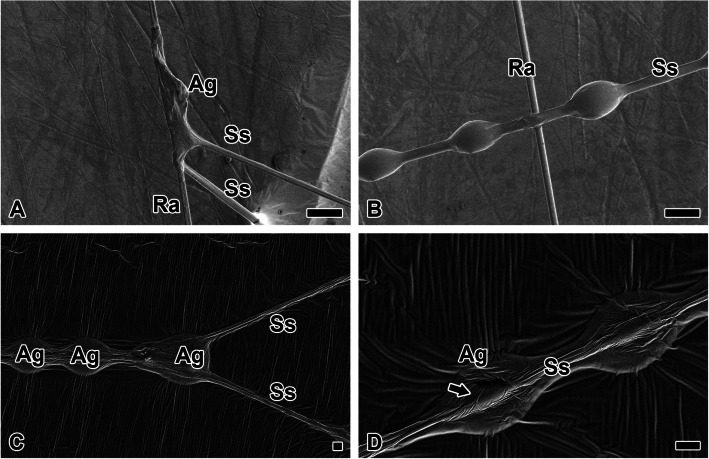
Structure of sticky spiral and glycoproteinaceous coating droplet. **A** Sticky spiral (Ss) is coated with glycoproteinaceous liquid silk secreted by the aggregate gland (Ag). In the hoof-shaped web, the radius does not spiral but instead forms loops at points slightly above the hub. **B** Artificially attaching radius (Ra) to sticky spiral did not result in strong bonding with brief contact alone. **C** During the looping, the sticky substance overlaps, creating larger droplets. These droplets can reach a diameter of approximately 100 μm. **D** Smaller droplets, around 65 μm in diameter, are observed on sticky spiral thread. At the center of each droplet, a condensed glycoprotein core is visible, enveloping a sticky spiral. All scale bars indicate 20 µm

Sticky spiral spinned from flagelliform silk gland serves as the actual spiral trap silk, containing gluey components that secure prey. The silk is coated with glycoproteinaceous liquid silk secreted by the aggregate gland (Fig. 6A). In the hoof-shaped web, the radius does not spiral but instead forms loops at points slightly above the hub. During directional changes, the spider does not undergo a separate silk-spinning process or wrap the radius to reverse direction. As a result, junction relies entirely on aggregate gland coating. Artificially attaching radius to sticky spiral did not result in strong bonding with brief contact alone (Fig. 6B).

During the looping, the sticky substance overlaps, creating larger droplets (Fig. 6C). These droplets can reach a diameter of approximately 100 μm. the sticky spiral silk bundles together into a single strand and then branches at regular intervals. Smaller droplets, around 65 μm in diameter, are observed on single strands of sticky spiral (Fig. 6D). At the center of each droplet, a condensed glycoprotein core is visible, enveloping a pair of sticky spiral silk fibers. Thick glycoproteinaceous coating envelops the entirety of the two pairs of sticky spirals.

**Fig. 7:**
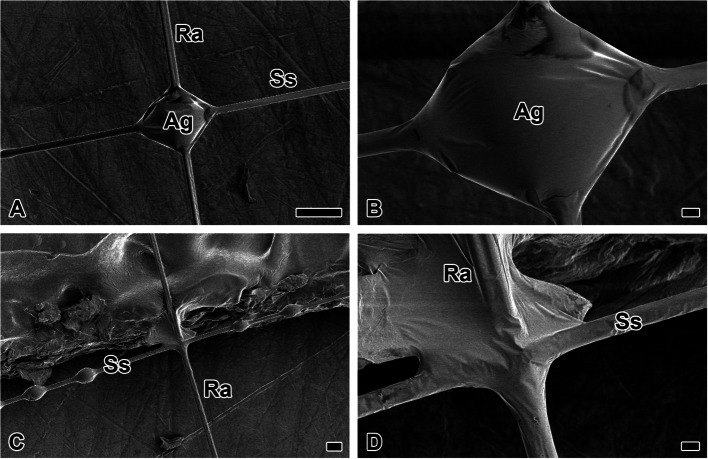
Adhesive interfaces between radius and sticky spiral. **A** The adhesive interface forming the lattice between radius (Ra) and sticky spiral (Ss) is reinforced by the accumulation of glycoprotein coatings (Ag). **B** The accumulated coating droplets are closer to a spherical shape than an ellipsoid, which typically coats a single silk strand. **C** The spherical coating droplets facilitate junction by maximizing the junction area between the radius and the substrate. **D** The two fibers intersect orthogonally without additional adhesion mechanisms. Each scale bar indicates 20 µm (A, C) and 5 µm (B, D)s

The adhesive interface forming the lattice between radius and sticky spiral is reinforced by the accumulation of glycoprotein coatings (Fig. 7A). The accumulated coating droplets are closer to a spherical shape than an ellipsoid, which typically coats a single silk strand (Fig. 7B). The spherical coating droplets facilitate junction by maximizing the junction area between the radius and the substrate (Fig. 7C). The two fibers intersect orthogonally without additional adhesion mechanisms (Fig. 7D).

**Fig. 8:**
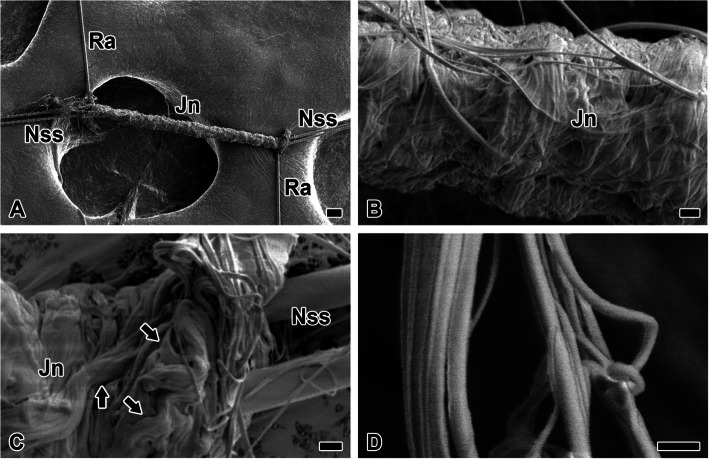
Junction attachment mechanisms and structural variations of minor ampullate and pyriform silk. **A** non-sticky spiral (Nss) exhibits significantly greater junction (Jn) with radius (Ra) compared to sticky spiral. The extended junction, exceeding 200 μm, generates considerable bending as the non-sticky spiral spirals around. **B** This junction is facilitated by pyriform silk (Py), which tightly wraps orthogonal structural silk with dense fibrils, obscuring the underlying structure. **C** Pyriform silk forms fibrils (arrow) as bundles, which perform coating functions. **D** Individual pyriform silk fibers are exceedingly fine, measuring less than 300 nm in diameter, with considerable thickness variation, exceeding a twofold difference even within the same fibril. Each scale bar indicates 20 µm (A, B), 2 µm (C) and 500 nm (D), respectively

Non-sticky spiral exhibits significantly greater junction with radius compared to sticky spiral (Fig. 8A). Localized structural folding along the spiral trajectory, facilitated by repeated 200 µm adhesive interactions with radius, enhances the web’s mechanical integrity. This junction is facilitated by pyriform silk, which tightly wraps orthogonal structural silk with dense fibrils, obscuring the underlying structure (Fig. 8B).

Pyriform silk forms fibrils (arrow) as bundles, which perform coating functions (Fig. 8C). Individual pyriform silk fibers are exceedingly fine, measuring less than 300 nm in diameter, with considerable thickness variation, exceeding a twofold difference even within the same fibril (Fig. 8D).

**Fig. 9:**
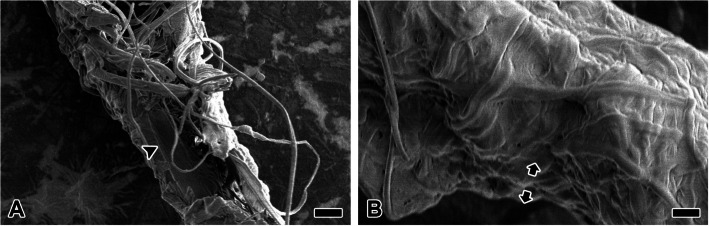
Structural organization and adhesion properties of pyriform silk coatings. **A** The pyriform silk coating layer that covers the radius and the non-sticky spiral is ruptured, revealing the internal surface (arrowhead). The internal surface appears smoother compared to the external surface, likely due to the adhesive coating substrate. **B** Meanwhile, the cross-section of the pyriform silk coating reveals approximately three layers of pyriform silk fibrils. This thickness, measuring around 1 μm, is sufficient to securely attach two structural silks without gaps. Each scale bar indicates 2 µm (A), and 1 µm (B)

The pyriform silk coating layer that covers the radius and the non-sticky spiral is ruptured, revealing the internal surface (Fig. 9A). The internal surface appears smoother compared to the external surface, likely due to the adhesive coating substrate. Meanwhile, the cross-section of the pyriform silk coating reveals approximately three layers of pyriform silk fibrils (Fig. 9B). This thickness, measuring around 1 μm, is sufficient to securely attach two orthogonal structural silks without gaps.

**Fig. 10:**
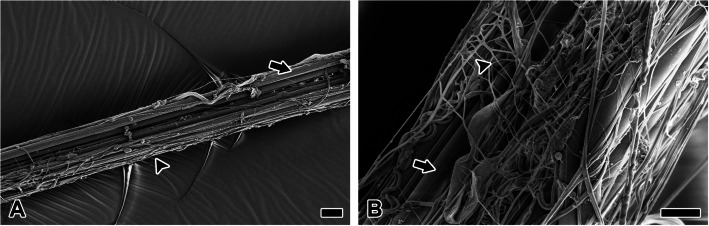
Bundles of structural silk supporting the orb web **A** The ampullate silk (arrow) progressively merge into bundled forms toward the edges of the web. Bundles lack significant pyriform gland adhesive coating (arrowhead). **B** While the bundles exhibit minimal coating along straight sections, adhesive coating layers are abundant at merging points. Each scale bar indicates 200 µm (A) and 50 µm (B)

The structural silk on both sides of the orb web is formed from ampullate silk. These silks secure three-dimensional junction points, enhancing the stability of the orb web plane. The complex structural silks progressively merge into bundled forms toward the edges of the web (Fig. 10A). While the bundles exhibit minimal coating along straight sections, adhesive coating layers are abundant at merging points (Fig. 10B). However, unlike the coating on the non-sticky spiral and radius of the orb web, the bundles are not entirely covered with a thick layer. Non-fibrous adhesive materials are scarce, while fibrous adhesive silks dominate.

**Fig. 11:**
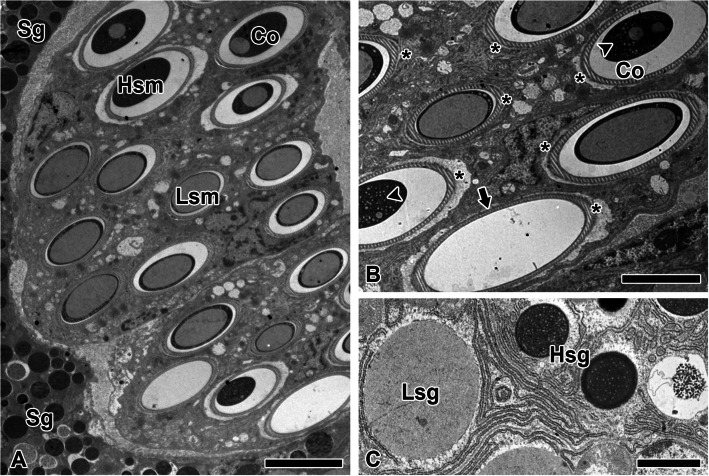
Secretory duct of the pyriform silk gland. **A** Two types of secretory materials were observed in the duct: a highly electron-dense secretory material (Hsm), presumed to be a cementing component, and a low electron-dense secretory material (Lsm), presumed to be a fibrous component. Sg: secretory granule. **B** The composite structure (Co) shows the coexistence of two secretory materials, with the highly electron-dense material surrounding the low electron-dense material. The low electron-dense material appears as densely distributed nanotubular structures (arrowheads). Although all ducts exhibit an identical membrane (asterisks), some ducts (arrows) lack passing secretions. The duct walls exhibit striated membrane-like structures. **C** Secretory granules are classified into highly electron-dense granules (Hsg) and low electron-dense granules (Lsg), both characterized by a well-developed rough endoplasmic reticulum. Scale bars represent 10 µm (A), 5 µm (B), and 2 µm (C), respectively

The cross-section of the pyriform gland reveals two distinct types of secretory substances (Fig. 11A). A large portion of the duct interior is actively involved in extruding the pyriform silk precursor. The highly electron-dense and low electron-dense materials correspond to the cement component and the fibrous silk precursor component, respectively. Additionally, a composite structure is observed in which the electron-dense cement encapsulates the low electron-dense silk material.

Secretory granules are distributed around the duct, and within the composite, nanotubular structures of the low electron-dense component are densely arranged (Fig. 11B). The proportion of the low electron-dense material within the composite varies, sometimes comprising the majority and other times appearing only in minor amounts. The secretory substances passing through the duct are discontinuous, as some regions of the duct lack visible material. Striated duct-lining membranes associated with cuticular layers were observed. No multilayered sheath or additional external structure was identified.

The secretory granules are distinctly divided into highly electron-dense and low electron-dense granules (Fig. 11C). The low electron-dense granules tend to exhibit a relatively larger diameter, while the highly electron-dense granules clearly display nanoparticle structures within their interior. Both types of granules are characteristic of typical secretory cells, exhibiting well-developed rough endoplasmic reticulum.

**Fig. 12:**
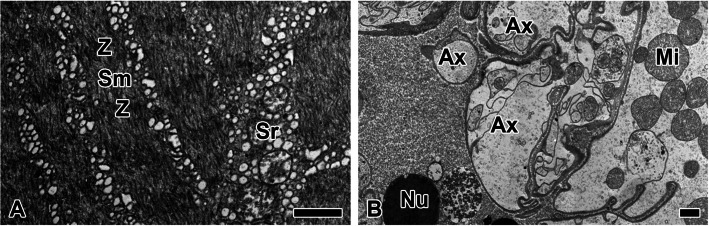
The muscular layer and neuromuscular junctions surrounding the pyriform duct within the anterior spinneret. **A** Myofibrils (white region) shows repeating units of sarcomere (Sm) which is defined by adjacent Z-lines (Z). Sarcoplasmic reticula (sr) are distributed around the myofibrils. **B** The peripheral region of the muscle cell layer is innervated by neural axons (Ax). The axons form neuromuscular junctions with muscle cells, enabling contraction and relaxation within the anterior spinneret. Nu: nucleus, Mi: mitochondria. All scale bars indicate 1 µm

Within the anterior spinneret, a muscle layer and neuromuscular junctions are observed. Myofibrils shows repeating units of sarcomere which is defined by adjacent Z-lines. Sarcoplasmic reticula are distributed around the myofibrils (Fig. 12A). The peripheral region of the muscle cell layer is innervated by neural axons (Fig. 12B). The axons form neuromuscular junctions with muscle cells, enabling contraction and relaxation within the anterior spinneret.

## Discussion

The golden orb-weaver spider, *Trichonephila clavata*, constructs a hoof-shaped orb web with the hub positioned at the upper part of the structure. In this design, both the non-sticky spiral scaffold and the capture spiral (produced by flagelliform gland) do not extend across the upper edge of the hub but instead form loops that cover a wide area of the lower section. Typically, the spider hangs inverted at the hub and utilizes gravity to swiftly capture prey positioned below. This preference for downward attacks is likely to become more pronounced as body size increases.

Indeed, spiders of the genus *Nephila*, including *T. clavata*, exhibit pronounced sexual dimorphism, with females observed to be significantly larger than other orb-weaving species (Kuntner and Coddington 2009). Members of this genus commonly construct expansive webs exceeding 1 m in diameter, with a broad hoof-shaped structure extending downward. Additionally, the permanent retention of the non-sticky spiral scaffold has been identified as a behavioral adaptation that enhances web stability and serves as a distinguishing feature of the genus *Nephila* within an evolutionary context (Kuntner and Agnarsson 2009).

The radius radiates outward from the hub, resulting in narrower intervals between the radii proximally and wider intervals distally. In *Trichonephila clavata*, this phenomenon is mitigated by splitting the radius, effectively equalizing the spacing. According to Kuntner (2006), such splitting occurs after the second or third turn of the non-sticky spiral within the radius domain. Kuntner (2007) further reported that these splitting can occur multiple times in nephilids, with as many as five instances observed.

Despite these compensatory efforts, we observed an increase in interval width toward the edges of the hub's lower region. This attempt to achieve uniform spacing may serve to efficiently distribute external forces, such as prey collisions, across the web while localizing damage (Blackledge et al. 2011). Eberhard (2014) suggested that similar branching in *Clitaetra perroti* supports the reinforcement of vertically elongated ladder webs in arboreal habitats.

The repair process of damaged spider webs may represent an extension of the same mechanisms involved in constructing split radii (Saravanan 2006). During web repair, the radius is restored first, forming the foundational structure. Tew et al. (2015) reported that spiders respond to weakened tension in radial threads rather than damage to the web itself during repair. Subsequently, the non-sticky spiral is woven in loops, effectively bridging the damaged area, and the process is completed with the reconstruction of the capture spiral (Cranford et al. 2012). This sequence mirrors the order observed during initial web construction, suggesting that the spider employs consistent behavioral patterns in both web formation and repair processes.

Web silk are predominantly composed of radius and non-sticky spiral, which are collectively known as dragline silk, serving as a lifeline for spiders (Xu and Lewis 1990). Specifically, dragline silk often consists of bundled fibers spun from both major and minor ampullate glands, whereas web silks display relatively less complexity in their composition. The non-sticky spiral consists solely of a pair of minor ampullate silk strands. The web’s structural silks are predominantly composed of either a single pair of major ampullate silk strands or a single pair of minor ampullate silk strands (non-sticky spiral). Radius, typically characterized as radius, was frequently observed in our electron microscopy analysis to lack the thickness commonly associated with major ampullate fibers. This observation aligns with findings previously reported by Blackledge et al. (2011). These insights highlight the necessity for further characterization of web structural silks to determine the specific ampullate gland origin of individual fibers in future studies. The distinct difference in composition between dragline silk and web silk lies in the fact that dragline silk is more directly tied to the spider's survival. It appears to have undergone evolutionary adaptations to maximize both flexibility and tensile strength (Craig 1987).

The junction between the radius and the sticky spiral consists solely of aggregate droplets, creating a simple contact-based connection, whereas the radius and the non-sticky spiral are joined by long and robust junctions formed by pyriform silk. The sticky spiral employs a sliding connection mechanism for its junction to the radius (Eberhard 1976). This connection method reflects the continuous nature of the capture spiral, as tension on one segment results in an adjustment of the thread distribution from adjacent segments, thereby increasing the length of the stressed segment (Blackledge et al. 2011). In this system, while the radius remains vertically fixed, the sticky spiral can slide laterally. When tension is applied, the stressed portion receives additional thread from neighboring segments, extending its length until the breaking threshold is reached. This adhesion system, requiring simultaneous forces on both sides to break, allows the web to withstand prey impacts with greater stability.

The junction between radius and the non-sticky spiral is mediated by pyriform silk fibrils, which wrap around both silks in multiple layers, forming a coating approximately 1 μm thick and extending over a length of about 200 μm. This robust junction, facilitated by pyriform silk, precludes the possibility of sliding connections and suggests that it is not intended for elongation under tensile stress caused by collisions (Wolff et al. 2015). On the other hands, pyriform silk does not directly contact the substrate surface but instead forms a thin and dense glue coating layer that mediates adhesion (Wirth et al. 2019). This tendency was also observed in the cross-section of the pyriform duct analyzed in this study. Most pyriform silk precursor groups passing through the duct exhibited a core structure composed of low electron-dense granules, presumably corresponding to spidroin, while the adhesive liquid encapsulated the outer layer. The inner surface of the pyriform coating appears smooth, likely due to the solidified adhesive liquid, while the outer surface exhibits a rough texture derived from the silhouette of pyriform silk fibrils (Wolff 2017). This duality in surface structure indicates that the adhesive interface maximizes contact with the ampullate silk substrate, firmly securing the silks without gaps (Grawe et al. 2014).

Pyriform silk gland secretions are composed of two primary components: silk proteins and a colloidal adhesive liquid (Sahni et al. 2012). The distal portion of the gland produces the silk, while the proximal region generates the adhesive liquid, both of which are co-secreted. The silk provides structural stability as a framework, and the adhesive liquid solidifies immediately after secretion, filling the interstitial spaces within the framework to ensure strong adhesion (Wolff et al. 2015).

The ratio of pyriform silk fibers to adhesive liquid in the formation of attachment discs has been reported to differ among spider species (Wirth et al. 2019). In *Nephila*, the amount of pyriform silk fibers is significantly higher compared to the adhesive liquid, unlike cribellate spiders, wandering spiders, and cobweb spiders. In the web of *T. clavata*, the substrates requiring adhesion by pyriform silk consist of two types of ampullate silk. Both substrates exhibit smooth, low-polarity surface structures, making the fiber-based composition of pyriform glue advantageous for achieving structural stability. This silk-and-adhesive complex wraps around the junction points between the radius and the non-sticky spiral, forming an adhesive coating.

Interestingly, spiders do not exhibit deliberate wrapping behavior while depositing the non-sticky spiral. Our results indicate that, unlike other silks, a layer of muscle cells is uniquely observed surrounding the pyriform silk gland. This layer is likely regulated by neurotransmitters from the spider's nervous system, enabling it to contract and potentially"push"the silk out for spinning. This muscle activity contracts the pyriform gland duct, facilitating the elongation and extrusion of the two types of secretory granules. Under applied pressure, a complex of the hydrophobic spidroin group and the adhesive liquid may form; however, they do not completely mix. Although a minor fraction of such complexes was observed within certain regions of the duct, the predominant structure consisted of the adhesive liquid encapsulating the fibers. This arrangement suggests that the low electron-dense granules, namely the adhesive liquid, exhibit hydrophilic properties.

Moon et al. (2012) observed approximately 200 spool-type pyriform silk spigots arranged around the ampullate gland spigots, potentially explaining the observed wrapping pattern of pyriform silk bundles. During junction, the spider elongates the contact area of the non-sticky spiral before applying the pyriform gland secretion. It is hypothesized that the broad, multidirectional wrapping of pyriform silk results from the elastic recoil of the elongated junction slip, causing the adhesive liquid and fibrils to intermingle and form a bundled structure. Notably, since the pyriform adhesive liquid exhibits hydrophilic properties, it likely encapsulates the hydrophobic silk fibers, contributing to interface stability during adhesion. Additionally, as elastic recoil occurs, the adhesive liquid may redistribute, enhancing the flexibility of the junction and modulating adhesive strength.

Observations of the *T. clavata* web reveal thick, golden-colored threads attached to external structures, primarily composed of barrier silk and frame silk bundled into dragline silk. Unlike the precise and robust connections formed between radius and the non-sticky spiral through extensive pyriform silk coatings, these dragline silk bundles resemble the minimal junction achieved by securing disordered wires with a cable tie. The lack of strong pyriform silk adhesion in these bundles suggests that external frame silks prioritize large-scale structural support and flexibility (Sahni et al. 2012). This contrasts with the localized tension distribution and web integrity ensured by the precise junction of radius and non-sticky spiral. While both utilize pyriform silk, the application scope and adhesion strategies appear to differ according to their respective structural roles (Craig 1997).

## Conclusion

This study provides a detailed microscopic analysis of the structural and adhesion mechanisms of the orb web in *Trichonephila clavata*, with particular emphasis on the role of the non-sticky spiral. The unique wrapping of pyriform silk fibrils, extending approximately 200 μm in length, underscores the importance of precise adhesion in maintaining web stability and resisting external forces. In contrast to the loose junction of the capture spiral, which relies on aggregate droplets for sliding connections and elongation, the non-sticky spiral junction demonstrates a commitment to structural integrity. These findings offer novel insights into the evolutionary adaptations and functional specializations of Nephilidae orb webs. Furthermore, the ecological significance of the often-overlooked non-sticky spiral is highlighted, with its adhesion mechanism enabling impact resistance and uniform tension distribution. These features present promising directions for future bio-inspired material applications.

## Data Availability

Materials described in the manuscript, including all relevant raw data, will be freely available to any scientist wishing to use them for non-commercial purposes.
